# Supplementary material for: High reliability organizations and healthcare safety outcomes on patients and staff: Scoping review
Source: PLOS Glob Public Health. 2026 Apr 3;6(4):e0006181. doi: 10.1371/journal.pgph.0006181 (PMC13048411; doi:10.1371/journal.pgph.0006181)
Supplement: Attachment — Submitted filename: Rebuttal Letter_Plos.pdf [file pgph.0006181.s002.pdf]

Dear Dr. Dhond and Editorial Team,

We sincerely thank you for your consideration of our earlier submission (Manuscript ID: PGPH-D-25-02316) and for the constructive feedback provided. We have carefully revised our manuscript to address the concerns noted in your decision letter. Below, we outline how we have responded to each point.

1. **Concern:** The literature review was completed over a year ago and is therefore not up to date.

   Response: We have fully rerun our literature search to include studies published between 2016 and 2025, ensuring that our review reflects the most current empirical evidence available on High Reliability Organization (HRO) implementation in healthcare.

We believe these revisions address the editorial concerns. We are grateful for the opportunity to revise and resubmit this work, and we hope that the updated version will now meet the journal's standards for publication.

Thank you for your time and kind consideration.

Sincerely,

Michael Joshua G. Morales, PhD, MHA, RN
School of Nursing
University of California, San Francisco
