# Supplementary material for: High reliability organizations and healthcare safety outcomes on patients and staff: Scoping review
Source: PLOS Glob Public Health. 2026 Apr 3;6(4):e0006181. doi: 10.1371/journal.pgph.0006181 (PMC13048411; doi:10.1371/journal.pgph.0006181)
Supplement: Attachment — Submitted filename: Line by Line Response to Reviewer.docx [file pgph.0006181.s003.docx]

Dear Editors,

Thank you for taking the time to review our manuscript and for the constructive and insightful comments provided by the reviewers. We greatly appreciate the opportunity to revise our work.

We have carefully addressed each comment and incorporated the recommended changes into the revised manuscript. We believe these revisions have improved the clarity, rigor, and overall quality of the paper. A point-by-point response to the reviewers’ comments is provided for your reference.

Thank you again for your thoughtful review and consideration of our revised submission.

Sincerely,

Authors

**Point by Point Response**

1. abstract: Add the information related to health care safety outcome and its important in introduction of abstract. Method section needs to mention electronic data searches to search for evidence, criteria used data extraction method etc. Include summary of findings of the studies along with associations. Revise conclusion

**Response:**
The abstract has been revised to emphasize the importance of healthcare safety outcomes, describe the electronic database searches, eligibility criteria, and data extraction methods, summarize key findings and associated relationships, and clarify the conclusion. Please see manuscript for revised abstract.

2. Methods: In search strategies, revise the last 3 line in the search strategies because these lines creates confusion in the inclusion of studies. Better to write studies beyond 2019 are also incorporated in this study as rationale

**Response:**

The Search Strategies section has been revised to clarify the rationale for the selected publication timeframe. The revised text now explicitly states that studies published beyond 2019 were intentionally incorporated to extend prior reviews and to capture more recent evidence on HRO implementation and outcomes, thereby reducing ambiguity regarding study inclusion. The revised section now reads:

*The search scope was limited to studies published in English between January 2016 and September 2025.* ***This time period was selected to incorporate relevant studies beyond 2019, extending prior reviews and capturing more recent evidence on the application and outcomes of HRO principles in healthcare.***

3. In inclusion and exclusion criteria, no need to repeat information in exclusion criteria that already mentioned in the inclusion criteria (1st 4 lines of 2nd paragraph)

**Response:**

To address the redundancy, we have removed the exclusion Criteria section, as it repeated information already specified in the inclusion criteria. This revision improves clarity and conciseness while maintaining the completeness of the eligibility criteria.

4. Data extraction and analysis: specify the data collection tool use in the study. mention about the validation of findings disagreement between two reviewer through discussion and with third person (.....)

**Response:**

We have revised the Data Extraction and Analysis section to clearly specify the use of the Joanna Briggs Institute (JBI) data extraction tool. We have also clarified that data extraction was performed independently by two reviewers, with any disagreements resolved through discussion and consensus. In addition, we have specified that a third reviewer was available to adjudicate unresolved disagreements; however, third-party arbitration was not required. These revisions improve the methodological transparency and rigor of the review.

5. Result: in result section, first paragraph is confusing. please write clearly about the publication of studies in chronological order. You have mentioned that 8 studies are conducted in US. Please mention the setting of other 2 studies also

**Response:**

The Results section has been revised to clearly describe the publication years of included studies in chronological order. In addition, the settings of all studies are now explicitly reported, noting that eight studies were conducted in the United States and the remaining two in Canada and New Zealand.

The revised text now reads:
*Ten studies included in this review were published between 2016 and 2025, with no studies published in 2020 or 2024 (Table 3). Geographically, eight studies were conducted in the U.S., indicating a concentrated application of HRO in this region. The remaining two studies were conducted in Canada and New Zealand, respectively.*

6. Discussion looks vague. Please revise your discussion with comparison of findings with other scooping and systematic review related to topics

**Response:**

The Discussion section has been revised to compare the findings of this scoping review with prior scoping and rapid evidence reviews on High Reliability Organization principles. The revised discussion situates the current findings within the existing literature, highlights similarities in the limited empirical evidence base, and clarifies how this review extends previous work by focusing exclusively on healthcare settings and by incorporating staff-related outcomes in addition to patient safety.

7. Conclusion: Revise your conclusion with emphasis of your study findings

**Response:**

The Conclusion section has been revised to place greater emphasis on the findings of this scoping review. Specifically, the revised conclusion highlights the observed impact of High Reliability Organization (HRO) practices on patient safety outcomes, including reductions in adverse patient safety events and improvements in patient safety culture. In addition, the conclusion now clearly identifies a gap in the existing literature regarding staff-related outcomes of HRO implementation, noting that only one study addressed this area, and emphasizes the need for further research.

8. Other: Repeated information in supportive information (26-31 and 1-6), error in page number, write the citation in PRISMA Flow, Specify the reason for the studies not retrieved in the PRISMA flow diagram (figure 1)

**Response:**

The repeated information has been removed, as it is supplementary in nature. Tables and figures are now uploaded in a separate file. Page number errors have been corrected, citations have been added to the PRISMA flow diagram, and Figure 1 now specifies the reason the studies were not retrieved.
